# The Role of Transposable Elements in Speciation

**DOI:** 10.3390/genes9050254

**Published:** 2018-05-15

**Authors:** Antonio Serrato-Capuchina, Daniel R. Matute

**Affiliations:** Biology Department, Genome Sciences Building, University of North Carolina, Chapel Hill, NC 27514, USA; gaserrat@email.unc.edu

**Keywords:** speciation, transposable elements, reproductive isolation

## Abstract

Understanding the phenotypic and molecular mechanisms that contribute to genetic diversity between and within species is fundamental in studying the evolution of species. In particular, identifying the interspecific differences that lead to the reduction or even cessation of gene flow between nascent species is one of the main goals of speciation genetic research. Transposable elements (TEs) are DNA sequences with the ability to move within genomes. TEs are ubiquitous throughout eukaryotic genomes and have been shown to alter regulatory networks, gene expression, and to rearrange genomes as a result of their transposition. However, no systematic effort has evaluated the role of TEs in speciation. We compiled the evidence for TEs as potential causes of reproductive isolation across a diversity of taxa. We find that TEs are often associated with hybrid defects that might preclude the fusion between species, but that the involvement of TEs in other barriers to gene flow different from postzygotic isolation is still relatively unknown. Finally, we list a series of guides and research avenues to disentangle the effects of TEs on the origin of new species.

## 1. Introduction

Speciation is the evolutionary process by which one lineage splits into two reproductively isolated groups of organisms [1]. One of the central goals of speciation research is to understand the processes that drive the evolution of reproductive isolation (RI) between species [2,3,4,5,6]. Significant strides have been made towards identifying barriers that generate RI between species [1,7], the processes underlying their evolution [2,3,8,9,10,11], and the rate at which they evolve during speciation [4,5,12,13,14,15]. Even though some progress has been made in identifying genes and loci associated with RI, few studies have explored the evolutionary processes that produced these barriers. Because of this, there is not yet a consensus as to what types of mutations or which mechanisms are typically involved in speciation or RI.

There are two broad approaches to identify the genetic underpinnings of RI. First, if crosses can be made, one can genetically map the loci underlying RI between organisms. Such studies can establish the genetic changes that maintain species identity and, if divergence is recent, potentially reveal the molecular changes that were initially involved in speciation. This approach is particularly informative when coupled with closely related organisms at different stages of reduced gene exchange [1,16,17]. An alternative approach is to assess whether a particular type of molecular change is commonly associated with isolation between genotypes. If a barrier to gene flow is commonly caused by a certain type of molecular change, then one can argue that that molecular change is important in either the origin of new species or the persistence of them when they face the possibility of collapse through gene flow. This approach has, for example, revealed that chromosomal inversions are commonly associated with the suppression of recombination and frequently harbor gene combinations involved in isolation between species [18,19] (reviewed in [20]). However, this approach has rarely been used to understand the impact of other molecular changes on RI. Here we highlight transposable elements as recurring agents that underlie a variety of manifestations of RI, which suggests they should be explored across various taxa in order to better understand their mechanistic and evolutionary contributions.

**Figure 1:**
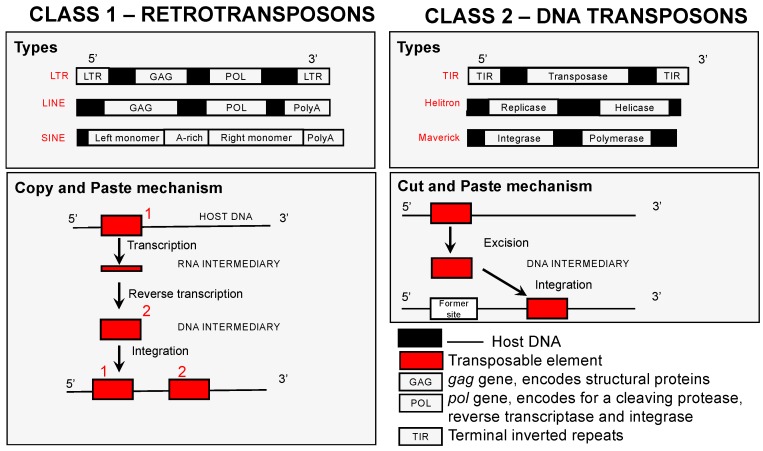
A graphical classification of transposable elements (TEs). The left panel shows Class 1 retrotransposons, and the right panel shows Class 2 DNA transposons. The upper panels show three examples of the genetic structure of each of these two classes of elements. The lower panels show the mode of movement (transposition mechanism) of each class. *LTR*: Long Terminal Repeats; *LINE*: Long interspersed nuclear elements; *SINE*: Short interspersed elements.

Transposable elements (TEs) are DNA sequences able to copy and insert themselves throughout the genome. TEs represent up to 80% of nuclear DNA in plants, 3–20% in fungi, and 3–52% in metazoans [21,22,23]. TEs are classified according to the mechanism they use to transpose. Class I elements require an RNA intermediate in order to integrate/duplicate themselves within a genome, while Class II elements act without an intermediate through a cut-and-paste mechanism that replicates its DNA directly to DNA as it mobilizes (Figure 1). A full classification of TEs is shown in Table 1. Interestingly, the predominant class of TEs can vary greatly between taxa [24,25,26,27,28] and species, and their genomic frequency, location, and activity levels can vary greatly even at the population level. TEs were described for the first time in maize by Barbara McClintock in 1950 [29] where they lead to somatic mutations affecting various phenotypes/genes depending on their chromosomal location and transposition time. The insertion of a TE can disrupt the coding or regulatory sequences of genes, which can cause deleterious effects by the modifying or eliminating a gene’s expression [30,31,32,33,34]. TEs are ubiquitous throughout nature [35,36,37] and their effect on their hosts’ fitness is generally considered to be deleterious; TEs are commonly considered selfish elements. However, gene disruptions are not the only consequence of TEs as they transpose throughout the genome. TEs can also cause regulatory changes, genomic expansions, and generate new chromosomal variants through the generation of inversions. Moreover, TEs can produce all of these changes rapidly [38,39,40] and in response to abiotic stressors—a hypothesis first advanced by McClintock [29]. These changes can provide genetic and phenotypic novelties upon which selection can act [41,42]. Due to their potential to generate novelty when it is needed, some have hypothesized that TEs are maintained in genomes through multilevel selection [43,44,45].

Thus, TEs are diverse and pervasive components of eukaryotic genomes that have the potential to impact rates of diversification and adaptation. TEs have also long been known to cause RI between genotypes (e.g., [78]). However, the role of TEs as a molecular mechanism capable of directly mediating the origin of new species remains underexplored experimentally.

The idea of selfish genetic elements and their involvement in the formation of new species has been latent in speciation genetics for years [90,91]. RI due to intragenomic conflict (i.e., conflictual speciation, reviewed in [92]) seems to be common but until recently was thought to be rare. Meiotic drive, endosymbionts, and maternal effects have all been implicated as potential sources of RI [1], and theoretical models have examined what role they may play in speciation [93,94]. Yet, the role of TEs in the initiation of the speciation process and in maintaining species has only rarely been experimentally studied. In this review, we highlight research that emphasizes TEs as important agents involved in the origin and persistence of species, with a focus the evidence for how TEs contribute to contemporary RI. We also propose future directions and questions that need to be addressed in order to understand whether transposable elements are involved in speciation, in the maintenance of species by generating reproductive isolation, and whether they cause distinct macroevolutionary dynamics.

## 2. Transposable Elements and Reproductive Isolation

Traits involved in keeping species apart can be classified depending on when they occur in the reproductive cycle. Premating barriers include ecological and behavioral traits that reduce the likelihood that two individuals will mate and include habitat isolation and mating choice. Post-mating-prezygotic barriers involve interactions between gametes and include sperm/pollen-egg incompatibility. Finally, postzygotic barriers arise after fertilization has occurred, and include various forms of fitness reductions in hybrids [1,3,95]. The genetic basis of prezygotic and postzygotic reproductive isolating mechanisms has been studied in varying degrees (reviewed in [1,95]), and a few studies have examined their connection to TE transposition (Table 2). In the following sections, we compile the cases for which TEs have been found to affect a trait potentially involved in RI, in an effort to emphasize their potential role as agents involved in various forms of reproductive isolation.

### 2.1. Premating Isolation I: Transposable Elements and Ecological Isolation

TEs have been hypothesized to promote local adaptation and enable the invasion of new habitats [96]. The initial colonization of a new environment is often accompanied by a reduction in genetic diversity as a result of genomic bottlenecks or founder effects. This hypothesis posits that by rapidly creating new genetic diversity, the transposition of TEs might help populations adapt to their new environment. Encountering a new environment is frequently stressful, and since TEs can be induced by stress TEs could facilitate an increase in genetic diversity exactly when it is needed [97,98,99]. The genomic shock model proposed by McClintock [29] that TEs mobilize in response to environmental challenges has been supported by many studies across multiple taxonomic groups [94,100,101]. New environments can select for different traits, and if these traits are associated with assortative mating (i.e., dual traits due to pleiotropy; [102,103]), then RI can evolve through divergent selection [104]. Therefore, we hypothesize that TEs could frequently underlie ecological adaptation and perhaps ultimately, speciation. A roadmap to assess whether local adaptation is commonly caused by TEs has been proposed elsewhere [96]. Notably, methods to detect TEs have evolved over the last five years and a fine scale dissection of the identity of the TEs in a genome and their copy-number throughout the genome is now feasible (Table 3), facilitating population level analysis. To examine evidence for our hypothesis, we focus on phenotypes that might lead to premating isolation and for which TEs have been shown to cause phenotypic differences.

Flowering time: Differences in flowering time are a common barrier to gene flow in angiosperms [105]. The mode of action is simple: differences in flowering time lead to RI between genotypes as the gametes of the two genotypes show a reduced probability of encountering each other. Additionally, changes in flowering time have several downstream effects that can further reduce the possibility of gene flow [106]. Besides the lack of contact of gametes due to the temporal differences, differing flowering time might also lead to differences in pollinators and thus fosters even stronger isolation than that caused by temporal differences alone.

In at least two cases, genetic mapping has revealed TEs underlying the disruption of genes involved in the pathways involved in flowering time and photoperiod.

The vegetative to generative transition 1 (*Vgt*, *ZmRap2.7*.1) locus in maize is an upstream (70 kb) noncoding regulatory element of a repressor of flowering. At *Vgt1*, a miniature inverted repeat transposable element (MITE) insertion into a conserved noncoding sequence was previously found to be highly associated with early flowering in independent studies [148]. The insertion of a *CACTA*-like transposon into the promoter of a second locus, *ZmCCT*, can suppress its expression through methylation and reduces maize sensitivity to photoperiod [149].

Similarly, in *Arabidopsis*, a recessive allele at the locus flowering locus C (*FLC*), is a result of disruptions of the gene by non-autonomous *Mutator*-like transposons, which ultimately leads to a delay in flower time. This transposon renders *FLC* subject to repressive chromatin modifications mediated by short interfering RNAs generated from homologous transposable elements in the genome [150].

TEs might play a role in floral induction and development in the rice shoot apex as a portion of them are silenced during floral induction [107]. The exact role these TEs play in floral induction is unknown, but the recurring activation and silencing of particular TEs, in particular *Gypsy* elements, at specific developmental stages suggests a regulatory overlap in reproductive development and TE produced small interfering RNA (siRNA). The downregulation of some retrotransposons stops them from repressing genes related to their transition into the reproductive phase, essentially activating genes required for flowering.

These examples show that TEs can modify the mean flowering time through a variety of mechanisms (e.g., differential methylation in maize, repression of an intron via siRNA in *Arabidopsis*). However, to our knowledge, all flowering time mapping cases have been done within species, thus far no case of between species difference in flowering time has been ascribed to TEs. It is worth noting that different molecular mechanisms resulting from TE insertion can produce the same phenotypic outcome. In maize, a TE insertion results in differential methylation in the regulatory region while in *Arabidopsis* there is repression at an intron through siRNAs. Interspecific differences in flowering time caused by TEs remain unidentified but it seems like a possible cause of isolation. 

Habitat isolation: Abiotic factors such as light and water availability can greatly influence the range over which a plant is able to spread, as well as influence the conspecific mates it will encounter. In the extreme, adaptation to a new environment can completely prevent contact with other members of a species, initiating the process of allopatric speciation.

The *CACTA*-like TE insertion (in the promoter for *ZmCCT*; [149]) implicated in photoperiod sensitivity in maize, has also facilitated local adaptation to temperate long-day environments. Additionally, variation in drought tolerance has been linked to a TE inserted in the promoter region of *ZmNAC111* [149]. This *MITE* insertion results in histone hypermethylation, which represses the expression of NAC resulting in a higher drought tolerance.

Selection acting across a continuous distribution of habitat preference can lead to RI as a byproduct of local adaptation to changing environmental factors. TEs may have generated the alleles selected during adaptation to temperate climates in *Drosophila melanogaster*. A study comparing temperature/latitudinal clines along Australia and North America found 10 TEs that show signs of positive selection at their insertion points, resulting in local adaptation [96,101,151]. By causing mutations in genes associated with a suite of traits, including circadian rhythm regulation and starvation resistance, several types of TEs (Long terminal repeats (*LTRs*), Long interspersed nuclear elements-like (*LINE*-like), and Terminal inverted repeats (*TIR*)) are thought to underlie the phenotypic differences along the cline. Suggestively, the TEs were more likely to be adaptive in temperate populations compared to tropical populations where they were likely to be neutral [151]. Taken together, these results strongly suggest that alleles generated by TEs were favored during local adaptation.

Host specificity in oomycetes: One of the main mechanisms of RI in plant pathogens is host specificity, which is regulated by the repertoire of effector genes within each pathogen. Effector proteins alter host physiology and allow colonization by individual pathogens [152]. In oomycetes, genomic distribution of TEs is frequently predictive of host specificity [153,154,155]. For example, the genome of *Phytophthora*, a major pest of commercial crops, harbors multiple families of retrotransposons (*copia*, *Gypsy/Ty*) [108,155,156,157,158]. In *Phytophthora infestans—*the potato blight pathogen—host specificity is regulated in part by RXLR class effectors that enable *P. infestans* to utilize a host [109]. As in other systems, TE insertion in *P. infestans* causes epigenetic silencing of both the transposon and nearby genes, resulting in regulatory differences. Notably, synthetic chimeras of a short interspersed element (*SINE*) to an effector gene in *P. infestans* leads to the silencing of both the introduced fusion and endogenous homologous sequences [109]. This silencing is also likely to occur naturally in the genome of *P. infestans*, as transcriptional inactivation of effectors is known to occur and over half of RXLR effectors are located within 2 kb of transposon sequences in the *P. infestans* genome. Thus, it is possible that host range in *P. infestans* was shaped by TEs inserted near these genes. Since mating in oomycetes occurs on host plants, it is plausible that TE insertions that modify host specificity have led to reproductive isolation in *P. infestans*. However, a systematic exploration of effector genes and their interactions with TEs would be needed to test this hypothesis.

### 2.2. Premating Isolation II: Transposable Elements and Sexual Isolation

Self-incompatibility: Fungi engage in diverse reproductive strategies, which often vary between closely related species [159]. Fungi often employ a mating system whereby the mating type—which is analogous to the sex—of the individual is determined by alternative alleles at one or several loci. In homothallic strains, which can mate with themselves (i.e., are self-compatible (SC)), additional loci generate allelic diversity at the mating type loci by a copy-paste mechanism (e.g., the homothallic switching (HO) endonuclease in *Saccharomyces cerevisiae*) [160,161]. Since single loci can effectively determine whether two individuals can interbreed or not, TEs can mediate transitions from homothallism (SC) to heterothallism (self-incompatible (SI)) in fungi by disrupting these loci. Transitions from self-incompatibility to self-compatibility are associated with speciation events (e.g., [162,163,164]) because selfing species are effectively isolated from other individuals and species (with the possible exception of somatic fusion; [112,165]).

Retroelements have contributed to the shifts from heterothallic ancestors to homothallic species in the *Neurospora* genus through mediating translocations at the mating-type (MAT) loci [166]. Retrotransposon insertions in the MAT locus also occur in *Blastomyces* and might be involved in decreasing the likelihood of recombination between mating types [167]. In other fungi, transposons have been found within or flanking MAT loci (e.g., *Neosartorya fischeri* [168], *Cryptococcus neoformans* [110], *Paracoccidioides brasiliensis* [167,169]), thus potentially providing an avenue for mating type to evolve independently through a rapid TE-induced mechanism. Specifically, in *Neurospora*, the transposition of *nsubGypsy* has facilitated the movement of genes neighboring the MAT loci to a different chromosome [165]. Transposition of *npanLTR* facilitates unequal crossovers between unrelated intergenic regions of opposite mating types, which in turn facilitates the transition into self-crossing species. Phylogenetic studies in *Neurospora* and *Kluyveromyces lactis* show multiple transitions from SI to SC species [166,170]. Taken together, these studies demonstrate that TEs may frequently be inserted at MAT loci, but it remains to be seen whether these patterns can be extended to other species.

Besides these effects on mating compatibility, genomic rearrangements mediated by transposition can also lead to viability issues in hybrids [171,172]. Barley rusts, *Ustilago hordei*, show a large increase in TE activity not observed in other closely related species (*Ustilago maydis* or *Sporisorium reilianum*), which has also led to both the reorganization of the MAT loci in the former species as well as large chromosomal rearrangements [173]. Few reports have explored a potential causal connection between TE activity and genome reorganization. A systematic assessment of how often TEs are involved in gene movement across chromosomes is sorely needed.

Transposons may also play an important role in transitions to self-compatibility in plants. *Solanum*, a flowering plant genus that contains tomatoes, consists of SC and (SI) taxa, with multiple transitions from self-incompatibility to self-compatibility [174]. SC taxa are characterized by low levels or no expression of stylar RNase (S-RNAse). The seven SC and the three SI taxa differ in the 5′ coding region of S-RNAse by several point mutations. Additionally, in one of the SI taxa, the source of low S-RNAse levels stems from an insertion of a transposon-like repetitive element. These results show how single-base mutations and the insertion of TEs can result in similar evolutionary outcomes [174].

These results suggest that transitions to self-fertility mediated by TEs might be common in fungi and plants. We hypothesize that since transitions to self-incompatibility have been associated with lower speciation rates and higher extinction rates in plants [175,176,177], TEs might be associated with differential diversification rates (i.e., species selection [178,179,180] in fungi. A formal test of this hypothesis remains to be performed.

Mating behavior in *Drosophila*: Behavioral isolation in *Drosophila* is mediated through a multimodal signaling system that involves cuticular hydrocarbons (CHCs), visual cues, and auditory signals [181,182,183]. CHCs are waxy compounds that are involved in desiccation protection (e.g., the species pair *D. serrata*/*D. birchii*; [184,185]) in the abdominal cuticle and are often necessary for mate discrimination and in some cases species discrimination [186,187,188,189]. Marcillac et al. [190] studied the effects of an insertion of a TE in the *desat1* locus and measured two different traits: the expression of CHCs and the ability of males to discriminate between the sexes. Even though no naturally occurring TEs have been found in the *desat1* locus, over 30 TEs have been found ~20–50 kb upstream of the gene [84,191]. *desat1* mutants (i.e., with a TE insertion) had lower CHC abundance (reducing the natural sex dimorphism) than lines without the TE. Moreover, mutant males showed poorer discrimination between control males and females suggesting that the TE insertion changed not only the emitted sexual signal but also how that signal is recognized. It remains to be seen if there are naturally occurring transposon-induced mutants in *desat1* or any other allele involved in the production of CHCs.

TEs have been conclusively shown to lead to interspecific differences in mating song in some *Drosophila*. Male flies in the *D. melanogaster* species subgroup produce a courtship song with two components: trains of continuous sinusoidal sound, called sine song, and pulses separated by an interval, called pulse song [27,192]. In the case of the sister species *Drosophila simulans* and *Drosophila mauritiana*, two species that diverged within the last 240,000 years [193,194,195], *D. mauritiana* males have a higher song frequency than *D. simulans* males, which in turn affects mating behavior and is a trait used by females to distinguish between conspecific and heterospecific males [196,197]. A retrotransposon, *Shellder*, has caused the disruption of the slowpoke (*slo*) locus in *D. simulans* [113]. The *slo* gene is expressed broadly in the fly nervous system and influences many locomotor behaviors and the insertion of *Shellder* leads to alternate splicing of the gene. *Shellder* insertions are polymorphic in their insertion sites in wild type strains of *D. simulans* and *D. mauritiana*, which strongly suggests that *Shellder* is probably propagating actively in *Drosophila* populations. The retrotransposon insertion seems to be polymorphic within *D. simulans*, which then leads to the question of whether this has led to isolation between different genotypes of *D. simulans*.

### 2.3. Transposable Elements and Postzygotic Isolation

TEs and chromosomal rearrangements: Chromosomal rearrangements are one of the genome features known to affect the likelihood of gene flow between species (extensively reviewed in [19,198,199,200]). In general terms, theoretical models indicate that chromosomal inversions can preclude gene flow at certain regions of the genome. Multiple empirical examples have shown that chromosome rearrangements can indeed contribute to postzygotic isolating mechanisms [201] and assortative mating [199], particularly when the rearranged regions contain alleles involved in reproduction. An active research program is trying to assess whether TEs can indeed lead to the origination of new chromosomal rearrangements (illustrated in [202] and reviewed in [66,67]). In *Drosophila buzzati*, the breakpoints of the *2j* inversion contain TEs. It has been hypothesized that *2j* might have originated by ectopic recombination of the TE at its breakpoints [114]. Even though this inversion has not been formally associated with RI, *2j* is involved with differences in life history traits among *D. buzzatii* populations [203,204]. The phenotypic effects of *2j* are contingent on genetic background, which suggests epistatic interactions with the rest of the *D. buzzatii* genome [115,205]. If TEs commonly induced inversions and other chromosomal aberrations, then TEs might play a role in maintaining species boundaries.

TE reactivation: In animals, fungi and plants, TEs are often targeted and silenced by siRNAs [206]. In plants, siRNAs involved in heterochromatin formation often target TEs and silence them [207]. Unlike animals, where the germ cells are formed early in development, plant germ cells differentiate from somatic cells in the adult and the chromatin remodeling ATPase decrease in DNA methylation 1 (*DDM*1) is crucial for this process. In *Arabidopsis,* *DDM1* is necessary to silence TE activity [206,207,208,209]. Even though TE reactivation and accumulation is restricted to the vegetative nucleus and not the sperm cells, TE accumulation in the vegetative nucleus can affect the sperm cells of the pollen and result in heritable changes [206]. In tobacco, just as in *Arabidopsis*, cytoplasmic connections between sperm cells and the pollen vegetative nucleus have previously been observed [206,210] and might provide a channel for siRNA and facilitate TE silencing. As a result, TE misregulation, which in essence is a hybrid specific defect of the TE-repressor system, might be a potential source of hybrid defects in pollen.

*DDM1* is also required to produce hybrid vigor (heterosis; [211]). *Arabidopsis* F1s between divergent accessions regularly show hybrid vigor in vegetative biomass throughout their lifecycle [212]. However, crosses involving *DDM1* loss-of-function mutants do not show heterosis; TEs are extensively expressed, which in turn causes abnormal and expression of genes related to salicylic acid metabolism [213]. Since fitness is so drastically affected by TEs, through either heterosis or hybrid incompatibility, these results might indicate that expression of TEs in hybrids changes their epistatic landscape (in a way that does not occur in pure species) with potentially deleterious effects. The role that *DDM1* plays in establishing RI could be tested by mutating *ddm1* across multiple plant lineages. The results from such mutagenesis approach will reveal whether this epigenetic regulator of TEs is involved in reproductive isolation in multiple species pairs.

Hybrid breakdown through deregulation of TEs is another postzygotic barrier between species. Lake whitefish lineages have repeatedly colonized postglacial lakes across North America. During these colonizations, a dwarf limnetic species has evolved from a benthic species multiple times. This repeated evolution has led to incomplete RI between the limnetic and benthic lineages [214,215]. Although the two lineages can produce viable hybrids, there is significant mortality in all hybrid types and backcrosses regularly show a malformed phenotype. Analysis of the transcriptome of hybrids reveals a 232-fold increase in TE activity in malformed embryos compared to pure crosses. This transcriptome wide deregulation of TEs results in shutdown of vital metabolic pathways drastically reducing the fitness of hybrids [216].

The reactivation of retroelements in hybrids can also lead to changes in chromatin profiles. Interspecific crosses of two Wallaby species, *Wallabia bicolor* and *Macropus eugenii*, produce hybrids with autosomes from *Macropus eugenii* that have a larger centromere [217,218]. The extended centromeres differ from those found in either parental species as hybrid centromeres consist primarily of un-methylated retrotransposons. TEs, then, can also affect chromatin structure and chromosomal composition in hybrids. Transpositions resulting from TEs being released from siRNA, epistatic, or epigenetic suppression mechanisms are pervasive across various eukaryotic groups and drastically change the fitness of hybrids.

Hybrid inviability: An extensively studied case of reproductive isolation is the genetic interaction between *Hmr*, *Lhr*, and *gfzf* in F1 hybrids between *D. melanogaster* females and *D. simulans* males. Alleles from these genes genetically interact to cause hybrid lethality between *D. melanogaster* and *D. simulans* [219,220,221,222]. RNA-seq analyses revealed that *Hmr* and *Lhr* are required to repress transcription from satellite DNAs and many families of TEs in their native hosts [222]. One possible cause of aberrant TE expression in hybrids is altered expression of *Piwi*-interacting small RNAs (piRNAs), a class of small RNAs that interacts with the *Piwi* family of Argonaute proteins to control the expression of TEs in the germline [223]. This is because the piRNA population in a host rapidly adapts, within ~6 generations [224], to the TE content through generation of new piRNA clusters, allowing de novo production of piRNA and other types of small RNAs for silencing of the invading TE [225,226]. Overexpression of TEs is frequently found in F1 hybrids, and is often associated with male sterility [227,228]. Overall these results suggest that the regulation of TEs might be of importance in maintaining contemporary species boundaries.

Hybrid dysgenesis: *Drosophila* is arguably one of the premier systems to understand the spread of TEs in animals. At least three families (*hobo*, *P*-elements, *I*-elements) have been found in *D. melanogaster* [68,229,230]. Of these families, *P*-elements (PEs) have received the most attention, as a result of a suite of defects in F1 hybrids (i.e., hybrid dysgenesis). Hybrid dysgenesis occurs in F1 hybrids from crosses between an uninfected female and an infected male [116,117], whereas individuals from the reciprocal cross are fertile. In dysgenic individuals, TEs proliferate and lead to a suite of defects such as chromosomal breakage, germ line cell apoptosis, and an increase in point mutations [78,231,232,233]. Despite drastic consequences PEs have spread throughout *D. melanogaster* [84,230,234] and *D. simulans* worldwide [235]. PEs are thought to have originated in the neotropical *D. willistoni* species group [79,236,237]. Although mites have been proposed to serve as a vector for PEs, potentially as a byproduct of their syringe-like feeding method [238], the precise mechanisms of this horizontal transfer remain unknown and untested.

The unidirectional development of hybrid dysgenesis between crosses stems from the way that genomes protect themselves the deleterious effects of PE activation. In F1 hybrid females, hybrid dysgenesis is only present in daughters from mothers with no PE and fathers with PEs. Usually the infertility that characterizes hybrid dysgenesis is silenced through piwi-interacting RNA silencing [239,240,241,242], which are exclusively maternally inherited. piRNAs seem to be present in all arthropods [243], and in the case of *Drosophila* piRNAs are cytoplasmatically deposited in embryos from females that contain PEs. Recent work shows that piRNAs are not alone in mitigating PE’s effects. PEs in *D. melanogaster* lead to hybrid sterility when the germoplasm does not carry the molecular machinery to regulate the expansion of PEs through minimizing cell apoptosis by co-opting the use of genomic maintenance genes such as *p53* [69].

A similar phenomenon, yet much less studied, occurs in *Drosophila virilis* [55]. The elements *Penelope*, *Ulysses*, *Paris* and *Helena* and *Telemac* have rapidly increased in frequency in natural populations. Experimental injection of *Penelope* causes germ line mutations as well as the activation of other TEs [55]. Similar to the hybrid dysgenesis phenomenon observed in *D. melanogaster*, when uninfected females are crossed to infected males, the resulting progeny show a high level of gonadal sterility, chromosomal nondisjunction and rearrangements, male recombination, and the occurrence of multiple visible mutations. There are however, notable differences between these two systems. While in *D. melanogaster* only one family of TEs are activated at once, in the *D. virilis* dysgenesis, all families are activated simultaneously [55,244]. The *Penelope* family seems to be primarily responsible for the hybrid dysgenesis syndrome of *D. virilis* [55].

If hybrid dysgenesis is a mechanism that can generate RI in populations of the same species, then the molecular machinery that regulates TEs might be important to not only maintain species boundaries at present but also facilitate speciation. This includes an assessment of whether TEs and TE-repressor system act as traditional genetic incompatibilities in hybrids [245]. A valuable research avenue will be to evaluate the effects of PEs in interspecific crosses and whether hybrid dysgenesis is a source of selection for speciation via reinforcement. 

Genomic imprinting in endosperm: Maturation of the embryo in angiosperms is contingent on normal development of the endosperm, a tissue that feeds the embryo during seed development [118,119]. Allocation of nutrients in the endosperm is consistent with parental conflict theory and excess dosage of paternal alleles promotes larger seeds while an excess of maternal alleles produces small seeds. This tissue is usually triploid and its normal development depends on the proper balance of gene imprinting [119]. Imbalances between paternally and maternally imprinted genes can lead to changes in gene expression through regulatory changes, a phenotype that is commonly aberrant in heterospecific hybrids (e.g., [246,247]).

*Arabidopsis arenosa* and *Arabidopsis thaliana* hybrid seeds show an overgrown endosperm and arrested or abnormal embryo development. *A. thaliana* harbors LTR retrotransposons of the *Ty3/Gypsy* family, known as *Athila*. These elements are large, with an internal region up to 10.5 kb long, flanked by an average of 1.8 kb LTRs on either side. This internal region produces two proteins: the *gag* capsid structural protein and *pol*, which carries the protease, reverse transcriptase and integrase domains essential for element duplication [248,249]. Seed inviability is positively correlated with the relative paternal genome dose, suggesting that maternal genomic excess suppresses incompatibilities in hybrids [246]. Moreover, the maternal genomic contribution (and thus seed viability) is inversely correlated with expression of *Athila* retrotransposons, expressed mostly from the pericentromeric regions. The normally silenced *Athila* (but not other TEs) is extensively expressed in hybrids. Only the paternal, and not the maternal, copies are expressed in these interspecific hybrids.

The precise reason why TEs are misregulated in hybrids relative to parentals remains unclear and likely varies across species. The interactions between paternally and maternally imprinted genes might lead to changes in silenced regions, which in turn is a common cause of postzygotic isolation in heterospecific crosses. Imprinting in plants is intimately associated with changes to methylation of TEs [120,250], and TE activity is known to alter DNA methylation patterns and gene imprinting in plant genomes [251,252,253]. Alternative molecular mechanisms—that might act in concert with perturbed imprinting—have also been proposed to account for seed failure, such as poor regulation of TEs by siRNAs in hybrids [254].

A systematic exploration of how often TEs promote post-zygotic isolation remains a promising research avenue to understand the link between TEs and speciation.

## 3. Introgression and Transposable Elements

Introgression, which is defined as the transfer of genetic material between species through the production of fertile interspecific hybrids, has recently been shown to be common across all domains of life [255,256]. Understanding what factors allow for gene exchange is crucial to understanding how species—especially nascent ones—persist in cases where they have the chance to interbreed and fuse into a single lineage. The relationship between transposable elements and introgression is multifaceted and includes (i) TE-aided introgression of non TE-DNA and (ii) interspecific transmission TEs alone.

**Figure 2:**
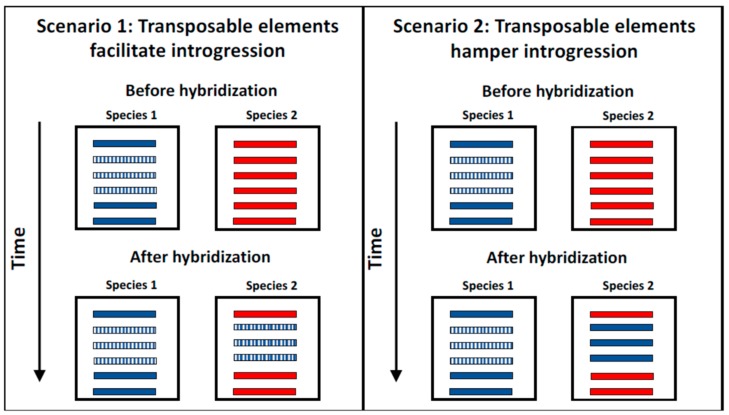
Two possible scenarios that illustrate potential connections between TEs and the likelihood of introgression. Two species are illustrated (blue and red). Stripped bars show chromosomes that contain TEs, while solid bars are chromosomes with no TEs. The left panel (Scenario 1) shows a potential scenario in which TEs facilitate the transfer of a full chromosome. The right panel (Scenario 2) shows a potential scenario in which TEs cannot cross the species boundary and thus chromosomes that harbor them are less likely to be introgressed. For simplicity only one direction of introgression is shown.

First, TEs might facilitate or hamper introgression of surrounding DNA. Surprisingly, this hypothesis remains untested even though its prediction is straight forward: if TEs increase the likelihood of introgression, then in hybridizing species regions that are TE-rich should show a larger amount of introgression compared to the rest of the genome. If, on the contrary, TEs hamper introgression through selection against regions containing TEs, then TE-rich regions should be refractory to introgression. These two scenarios are illustrated in Figure 2. Even though no systematic study has addressed whether TEs facilitate introgression, there are some indications TEs might be involved in horizontal gene transfer (HGT) [257,258]. The coffee berry borer beetle, *Hypothenemus hampei* [259], and the mustard leaf beetle, *Phaedon cochleariae*, appear to have acquired the genes necessary for their specialized diet through a HGT from bacteria [260], allowing them to degrade plant cell walls. Interestingly in both cases, the genes acquired by the beetles are flanked by two transposons. The potential role TEs might have played in this transfer remains suggestive but inconclusive.

Introgression might also lead to the transfer of TEs across species boundaries [261,262]. HGT have been linked to speciation events (or at least specialization events) in bacteria, providing novel gene sets that expand host specificity. Horizontal gene transfer regularly acts as a genetic bridge between vastly diverged species [257,258]. Horizontal transfers of TEs between angiosperm genomes have been documented in nature [263,264,265] and experimentally [266]. In *Drosophila*, HGT seems to have occurred from the *willistoni* species group to *D. melanogaster*. The two groups diverged over 50 million years ago and there is no possibility of hybridization [267]. Many other cases of HGT between species (with a rapidly growing list) have also been reported but the precise mechanisms of gene exchange remain largely unknown and might differ between taxa and reproductive strategies [118,268,269]. By serving as a pathway to TE acquisition, HGT can result in RI when coupled with the effects of new TEs entering a genome.

The most likely mechanism of transfer of genetic material between closely related species is arguably the production of fertile hybrids with subsequent introgression. Even though it is clear that TEs can be mobilized by HGT, it remains unclear to what extent TE activation can occur through introgression. This question remains largely unexplored both in natural and experimental populations. This scarcity is puzzling because the proposal that introgression mediated by hybridization could lead to transposon introduction and mobilization within the genome of rice is not new (i.e., a genome shock, [270]). Two examples of TE mobilization following introgression stand out. First, recombinant inbred lines produced by hybridizing rice species (cultivar Matsumae and wild rice *Zizania latifolia*) have shown that the miniature-Ping (*mPing*) TE together with its putative transposase-encoding partner, Pong, can be mobilized between species [271,272]. Likely, the mobilization of mPing and Pong is a result of introgression-induced malfunction of the established cellular control systems in the rice genome, as their transposition is transitory and rapidly repressed.

The second example comes from experimental hybrid swarms between two divergent species of *Drosophila*: *D. melanogaster* and *D. simulans*. Both species harbor the *Bari-I* element, a Class II TE with an open reading frame able to encode a polypeptide with 339 amino acids. (The sequence of the putative protein in *Bari-I* is similar to the transposase of the *Tc-1* element of *Caernorhabditis elegans*, which might in turn suggest HGT across animal orders [273].) In synthetic hybrid swarms using *D. simulans* C167.4, an unusual line that produces fertile hybrid offspring with *D. melanogaster*, *Bari-I* elements, originally from the *D. melanogaster* parent, are maintained in hybrid strains, suggesting that introgression can indeed be a mechanism of transfer of TEs. The element is present across the geographic range of both species and shows such similar sequence that it seems to be transmitted horizontally and not vertically [274].

Introgression of TEs has been hypothesized for *Drosophila bifasciata* and *Drosophila imaii* [80], species of the *simulans* complex [275], species of the groups *willistoni* (reviewed by [276]), *saltans* ([277,278]), and the species pair *Drosophila serido* and *D. buzzatii* [279]. The main lines of evidence in these studies have been the ability of these species to produce fertile hybrids and the sequence similarity of TEs across species [72,80,280,281]. A full and detailed characterization of the rates and nature of introgression awaits for most of these groups and should be coming in the near future as TEs will continue to be a focus of research due to their diverse effects across organisms.

## 4. Future Directions

The relationship between TEs and RI is an open field of research that will likely increase in prominence over the next few years. Given the broad range of roles TEs have played in affecting gene exchange between various species, further study is required in order to better understand the extent to which TEs influence evolution and speciation. Box 1 lists focal questions that remained unanswered. These questions fall into three broad categories.

### 4.1. Are Transposable Elements a Common Cause of Reproductive Isolation?

Mapping the precise genetic basis of interspecific differences will reveal what type of mutations and genomic interactions are more likely to cause and maintain interspecific differences and their relative contribution to various forms of RI. This will lead to a better assessment of the relative importance of TEs as a genetic cause of RI. A second line of research will explore the role of TEs in adaptation to the peripheral areas of geographic range of a species. In maize, for example, *Mutator* TEs are reactivated in response to environmental stress [282], which is most likely to occur at the edge of the optimal range of the species. TE reactivation might induce to genomic changes that in turn lead to RI between peripheral populations in extreme environments and the central populations’ (akin to peripatric speciation; [1,283]). Moreover, hybrid zones are usually found at the edges of the range of the hybridizing species so the interplay of hybridization and potential activation of TEs due to environmental or competition induced stress should be examined (Questions 1–5 in Box 1).

### 4.2. Are Transposable Elements Responsible for Differential Rates of Diversification?

The broad range of genome sizes across eukaryotes is partially explained by the quantity of repetitive, non-coding DNA—including TEs—interspersed throughout the genome [22,118,284,285,286,287,288]. The consequences of genome expansions are significant and have been linked to the duration of meiosis, ecological distribution, speciation rate, and extinction risk (e.g., [289,290] reviewed in [291]). Genome rearrangements and, in particular, genome duplications have been associated with higher rates of diversification in teleosts [292,293,294] and angiosperms [295,296]. The reasoning behind why genome duplications lead to an increase in diversification rates remains unclear but generally there are two explanations. First, genome duplication allows for gene subfunctionalization and neofunctionalization that would not be possible in a non-duplicated genome [297,298,299,300]. Second, large genomes might simply have the chance to accumulate more hybrid incompatibilities. Only one systematic evaluation of the relationship of genome size and cladogenesis has been performed (for angiosperms) and it found evidence of a positive correlation between overall genome size and rates of speciation [301,302]. Since TEs commonly lead to an increase in genome size, this is consistent with the hypothesis that invasion by TEs can increase the rate of speciation.

An evaluation of this hypothesis has been carried out in haplochromine cichlids. A comparative analysis to determine what traits were correlated with successful adaptive radiations in Lakes Malawi and Victoria found that traits like decoupled pharyngeal jaw and maternal mouth brooding—which have been hypothesized to be key innovations enabling diversification in cichlids—could not account for differences in the rate of diversification in this group. In contrast, increased numbers of *SINE* insertions preceded the extensive radiations within each lake [303]. These results are consistent with TEs mediating adaptation through either gene disruption or altered methylation patterns near insertion sites. However, determining whether TEs generally lead to increased speciation rates will require a formal macroevolutionary test in which the sample size (i.e., potential radiations caused by TEs) is larger than one [304].

Conversely, TEs could result in increased extinction rates and therefore lead to decreased diversification rates. This is related to an intriguing hypothesis that posits that asexual groups form reciprocally monophyletic clusters (akin to asexual species) rapidly but also disappear rapidly due to the proliferation of deleterious transposons inherited from their sexual progenitors that cannot be purged by recombination, leading to extinction [42,305]. (Questions 6–9 in Box 1) Additional studies are required to move conclusively test a possible connection between TEs and diversification rates.

### 4.3. Are Transposable Elements and Hybrid Dysgenesis a Source of Selection for Reinforcement?

Hybrid dysgenesis is a phenomenon that occurs in animals and might also exist in plants. Even though its natural frequency remains currently unknown, it is possible that it might be rather common [243,244]. Similarly, reinforcement, the evolutionary process in which prezygotic isolation is strengthened as a byproduct of the production of unfit hybrids, seems to be pervasive in nature [306]. If F1 interspecific hybrids consistently suffer fitness defects due to hybrid dysgenesis, then natural selection might indirectly penalize individuals that mate with heterospecifics, thus fostering the completion of speciation (i.e., increasing RI until there is cessation of gene flow). This question also remains unanswered and will require the identification of sister species that hybridize in nature and for which hybrid dysgenesis represents a major cost to heterospecific mating (Questions 10–11 in Box 1).

Box 1 **Unanswered questions about the connection between TEs and speciation.**

1. Are interspecific differences in flowering time disproportionately caused by TEs?
2. Do transposons play a significant role in pathogen adaptation to new hosts?
3. How commonly are TEs involved in antibiotic resistance?
4. How is the likelihood of chromosomal inversions caused by recombination affected by TEs?
5. What is the role of TEs in causing hybrid breakdown?
6. Do TEs regularly mediate the transition from outcrossing to self-crossing in fungi?
7. What is the taxonomical distribution of TEs?
8. Do TEs cause changes in the net rates of diversification across the tree of life?
9. Can TEs be deleterious enough to cause extinction?
10. Is introgression facilitated or hampered by TEs?
11. Does hybrid dysgenesis facilitate speciation by reinforcement?
12. Can TE-repressor systems generate hybrid incompatibilities during speciation?

## 5. Conclusions

Transposable elements are hypothesized to promote bursts of diversification or biological and genomic differentiation between species (e.g., [94,99]). Yet there is little direct evidence that TEs can indeed facilitate RI and ultimately speciation. That does not mean TEs are not related to the generation of new genetic elements, genetic circuits, and ultimately of phenotypes. On the contrary, TEs are commonly associated with the origin of new genetic and phenotypic diversity through gene regulatory element innovation, genic disruptions, siRNA/epigenetic suppressor mismatches, and chromosomal remodeling. In vertebrates, TEs have regularly contributed to the evolution of regulatory and coding sequences, leading to new lineage-specific gene regulations and functions. Their role has been pivotal to generate new phenotypic diversity. In primates for example, TEs are the main source of new variants in regulatory sequences [307]. In angiosperms a significant portion of adaptive novelty is due to the activity of TEs (active TE-Thrust), resulting in an extraordinary array of genetic changes, including gene modifications, duplications, altered expression patterns, and exaptation to create novel genes, with occasional gene disruption [118]. Even though it is clear that TEs are involved in generating the genetic material for new traits (some of them involved in adaptations), the question of whether TEs are involved in RI has remained largely understudied. The combination of natural history, genetics and genomics will reveal the prevalence of TEs in nature and to what extent they have played a role in generating and sustaining new organismal diversity.

## Figures and Tables

**Table 1:** A classification of the different types of transposable elements.

Type	Name	Activity	Taxonomic Distribution	Insertion Preference	Function/Pathway Influenced	Citations
Retrotransposons (class 1)					Replicate through reverse transcription of an mRNA intermediate, the resulting cDNA product integrates	
Long-tandem repeats						
	*BEL/Pao*-like elements	non-autonomous	Metazoans	Undescribed	Second most abundant retrotransposon but very little is known.	[46,47]
	*DIRS1*-like retrotransposons	autonomous	Common in decapods, sparse among other Eukaryotes	Preferentially integrates into other DIRS-1 sequences and GTT sequences	Undescribed	[48,49]
	*Ty1/copia*	autonomous	Eukaryotes	Preference towards upstream region of RNA Pol III, near tRNA genes	Mutational agent and can mediate genome rearrangement through recombination.	[50,51]
	*Ty3/gypsy*	autonomous	Eukaryotes	Upstream of RNA polymerase III transcription, near tRNA genes	Mutational agent and can mediate genome rearrangement through recombination.	[50]
	*Ty5*	non-autonomous	Fungi	Integrates near areas of silent chromatin at the telomeres and mating loci	An increase in recombination at insertion points	[52]
Non-LTR						
	*Alu*	non-autonomous	Primate specific	Fixed at C-terminus of Human HPK1 and throughout genome	Cause insertion mutations, increase recombination, change gene expression through gene conversion	[53]
	*LINE* (long interspersed nuclear elements: *Jockey*, L1, L2, R2)	autonomous	Eukaryotes	R2 inserts into 28S ribosomal DNA genes but has a strong bias against previous R2 insertions.	Encodes proteins responsible for packing of RNA transcript and a polymerase that enables reverse transcription, with an endonuclease subsequently integrating it into the genome.	[54]
	*Penelope*	autonomous	Metazoans, rare in Plants	Insertions of element have been linked to breakpoints in inversions within *D. virilis*	Element that underlies hybrid dysgenesis in *D. virillis*.	[55,56]
	*RTE* (RNA transport element)	non-autonomous	Metazoans	Do not appear to be sequence specific	Upon insertion has been shown to result in target site duplications	[57,58,59]
	*SINE* (short interspersed nuclear element)	non-autonomous	Plants, metazoans, fungi	Bias against insertion in intronic splice sites and preferentially inserts into the 3′ region of introns	Shown to control mRNA production and repress transcription of protein coding genes	[60,61]
	*VIPER/Ngaro*	autonomous	Metazoans, fungi	Undescribed	Undescribed	[62]
Transposons (class 2)					Replicate through a DNA intermediate	
	*CACTA*	autonomous	Plants	Located near centromere	Results in increased methylation and structural changes between genetic orthologs	[63]
	*Crypton*	autonomous	Fungi, arthropods	Unknown	*Crypton*-derived genes function as transcriptional regulators	[64]
	*Helitron*	autonomous	Plants, metazoans, fungi	Preferentially inserts in gene-rich regions	Ability to capture gene sequences, including introns.	[65]
	*hobo*	autonomous	Arthropods	Biased towards areas with high recombination rate	Can mediate recombination and inversions	[66,67,68,69]
	*I-element*	autonomous	Plants, metazoans	Located near centromere heterochromatin	Transpose in germline at a high rate and are repressed maternally	[70,71]
	*Mariner/Tc1*	autonomous	All groups	Associated with heterochromatin	Provide a hotspot of recombination in *Drosophila* females	[62,72,73,74]
	*Mavericks/Polinton*	autonomous	Eukaryotes, some prokaryotes	Unknown	Retrovirus-like and codes its own DNA polymerase	[75,76]
	*Mutator*	autonomous	Plants	Insertions concentrate in epigenetically marked open chromatin	Insertion sites are correlated with recombination rates	[77]
	*P-element*	autonomous	Plants, metazoans	Insert at random with a preference for 5′ untranslated regions	Underlies hybrid dysgenesis and greatly increases mutation rate	[78,79,80]
	*PIF-Harbinger*	autonomous	Plants	Target site preference for TAA	Insertion into regulatory genes resulted in pigmentation changes in maize	[81]
	*piggyBac*	autonomous	Metazoans	Throughout the genome	Acts as an insertional mutagen.	[82,83]
	*pogo*	autonomous	Metazoans	Likely to insert in regions with low denaturation temperature	Often leads to deletions	[84,85]
	*Rag-like*	autonomous	Metazoans	Undescribed	Linked to recombination and affects immune system response	[86,87]
	*Transib*	autonomous	Eukaryotes	Undescribed	May underlie the development of new genes	[88,89]

**Table 2:** A summary of reproductive isolating barriers for which TEs have been invoked as a potential cause. A full description of the involvement of TEs is presented in the text. Stars represent cases that remain suggestive but for which more evidence is required (see text).

Type of Reproductive Isolation	TE-Mediated Phenotype	Examples and References
Premating isolation	Adaptation to new habitats.	Flowering time [63,107] Host specificity [108,109]
	Insertions at loci that control self-compatibility.	Shift of reproductive strategies lead to reproductive isolation [110,111] TE movement can lead to gene movement and aneuploidy in hybrids [112]
	Changes in traits involved in recognition of conspecifics.	Mating song frequency between sibling species [113]
	Changes in genome structure.	TE-induced chromosomal inversions [114,115]
Postzygotic isolation	Hybrid sterility as a result of reactivated transposition.	Hybrid dysgenesis [55,69,116,117]
	Misregulation of TEs leading to hybrid inviability	Overgrown endosperm; abnormal embryo development [118,119,120]

**Table 3:** Computational methods to detect transposable elements using genomic data.

TE Detection Tool	Year	Language	Reference
MELT	2017	Java	[121]
IT IS	2015	Perl	[122]
Jitterbug	2015	Python	[123]
DD_DETECTION	2015	C++	[124]
TIDAL	2015	Perl, R	[125]
Mobster	2014	Perl	[126]
Tangram	2014	Java	[127]
T-lex2	2014	Perl	[128]
TIF	2014	Perl	[129]
TranspoSeq	2014	Java, R	[130]
TraFiC	2014	Perl	[131]
TIGRA	2014	C++	[132]
TE-Tracker	2014	Perl	[133]
GRIPper	2013	Python	[134]
RelocaTE	2013	Perl	[135]
Tea	2012	R	[136]
ngs_te_mapper	2012	R	[137]
TE-Locate	2012	Java, Perl	[138]
REPET	2011	Python	[40]
VariationHunter	2010	C++, Python	[139]
HYDRA-SV	2010	C++, Python	[140]
MITE-Hunter	2010	Perl	[141]
SeqGrapheR	2010	R	[142]
RISCI	2010	Perl	[143]
MoDIL	2009	Python	[144]
LTRharvest	2008	C	[145]
HelitronFinder	2008	Perl	[146]
TransposonPSI	2008	Perl	[147]
